# Brain volumetric and metabolic correlates of electroconvulsive therapy for treatment-resistant depression: a longitudinal neuroimaging study

**DOI:** 10.1038/tp.2016.267

**Published:** 2017-02-07

**Authors:** M Cano, I Martínez-Zalacaín, Á Bernabéu-Sanz, O Contreras-Rodríguez, R Hernández-Ribas, E Via, A de Arriba-Arnau, V Gálvez, M Urretavizcaya, J Pujol, J M Menchón, N Cardoner, C Soriano-Mas

**Affiliations:** 1 Department of Psychiatry, Bellvitge University Hospital-IDIBELL, Barcelona, Spain; 2 Department of Clinical Sciences, School of Medicine, University of Barcelona, Barcelona, Spain; 3 Magnetic Resonance Department, Inscanner SL, Alicante, Spain; 4 Carlos III Health Institute, CIBERSAM, Madrid, Spain; 5 Mental Health Department, Parc Taulí Sabadell, Universitat Autònoma de Barcelona, Barcelona, Spain; 6 School of Psychiatry, University of New South Wales (UNSW) and Black Dog Institute, Randwick, Sydney, NSW, Australia; 7 MRI Research Unit, Radiology Department, Hospital del Mar, Barcelona, Spain; 8 Department of Psychobiology and Methodology in Health Sciences, Universitat Autònoma de Barcelona, Barcelona, Spain

## Abstract

Recent research suggests that neuroplastic and neuroinflammatory changes may account for the mode of action of electroconvulsive therapy (ECT), although extant data do not allow for a clear disambiguation between these two hypotheses. Multimodal neuroimaging approaches (for example, combining structural and metabolic information) may help in clarifying this issue. Here we aimed to assess longitudinal changes in (i) regional gray matter (GM) volumes and (ii) hippocampal metabolite concentrations throughout an acute course of bitemporal ECT, as well as (iii) to determine the association between imaging changes and clinical improvement. We assessed 12 patients with treatment-resistant depression (TRD) at four time points (pre-treatment, after the first ECT session, after the ninth ECT session and 15 days after ECT course completion) and 10 healthy participants at two time points, 5 weeks apart. Patients with TRD showed bilateral medial temporal lobe (MTL) and perigenual anterior cingulate cortex volume increases. Left MTL volume increase was associated with (i) a hippocampal *N*-acetylaspartate concentration decrease, (ii) a hippocampal Glutamate+Glutamine concentration increase and (iii) significant clinical improvement. The observed findings are, in part, compatible with both neuroplastic and neuroinflammatory changes induced by ECT. We postulate that such phenomena may be interrelated, therefore reconciling the neuroplasticity and neuroinflammatory hypotheses of ECT action.

## Introduction

Electroconvulsive therapy (ECT) is a well-established alternative for treatment-resistant depression (TRD).^1^ Nevertheless, its mechanism of action remains poorly understood. Recent studies have used magnetic resonance imaging (MRI) to explore the changes in brain structure putatively associated with ECT and have consistently reported volume increases in limbic regions such as the hippocampus, the amygdala or the anterior cingulate cortex (ACC).^2, 3, 4, 5, 6, 7, 8, 9, 10, 11, 12^

Although recent reports advocated that such ECT-induced structural changes could result from neurogenesis induction in neurogenic regions (that is, dentate gyrus^13^), the extension of volume increases to other hippocampal subfields and limbic regions^14^ suggests that they are likely better understood as resulting from more general structural neuroplastic changes, which embrace different molecular mechanisms in addition to neurogenesis, such as synaptogenesis, gliogenesis or angiogenesis.^15^ Likewise, other reports suggested that ECT-induced structural changes might partially depend on neuroinflammatory mechanisms.^8, 16^ It has been shown that ECT may increase the permeability of the blood–brain barrier,^17^ possibly leading to a local swelling of adjacent brain tissue (that is, vasogenic edema).^16^

The assessment of brain regional metabolite concentrations may complement structural studies in ascertaining the neurobiological correlates of ECT. Magnetic resonance spectroscopy (MRS) studies have shown both decreases and increases of *N*-acetylaspartate (NAA) across different regions, such as the ACC, the dorsal lateral prefrontal cortex or the amygdala after ECT.^18, 19, 20^ Moreover, four studies have explored ECT-induced NAA changes in the hippocampus,^9, 20, 21, 22^ finding decreases^20^ or no changes^9, 21, 22^ in NAA concentrations. At the hippocampal level, increases in Cho (choline-containing) compounds have been also detected in two studies.^21, 22^ However, despite increases in Glutamate+Glutamine (Glx) concentrations could result in neuronal swelling and excitotoxicity,^23^ which might support the neuroinflammatory hypothesis of ECT action, this metabolite has been only assessed in one previous study at the hippocampal level, where a decrease in Glx concentrations after ECT was observed.^20^ Conversely, significant Glx increases after ECT have been observed in the ACC, the dorsal lateral prefrontal cortex or the amygdala.^18, 20, 24, 25^

In addition, from a clinical perspective, such neurobiological mechanisms allegedly accounting for ECT effectiveness have been ill-related with clinical outcomes. For example, only two studies have presented findings directly relating structural changes with the symptomatic improvement of TRD patients. In these studies, gray matter (GM) volume increases in the hippocampus^10^ and the amygdala^7, 10^ were associated with symptom improvement after ECT. Moreover, only one MRS study has reported significant associations between hippocampal Glx concentration decreases and subgenual anterior cingulate Glx concentration increases and clinical improvement.^20^ In the present study, to better characterize the neurobiological correlates of ECT, we performed a longitudinal assessment of TRD patients throughout an acute course of bitemporal ECT (that is, pre-treatment, after the first ECT session, after the ninth ECT session and 15 days after ECT completion). Clinical and imaging (structural MRI and MRS) measurements were obtained in each time point. The specific aims of the study were: (i) to assess longitudinal changes in regional GM volumes, (ii) to investigate longitudinal changes in hippocampal metabolite concentrations and (iii) to study the association between imaging changes and clinical improvement. On the basis of the previous literature, we expect to find regional volume increases in limbic structures after ECT. We also anticipate that hippocampal metabolite concentrations (specifically, NAA and Glx) will change after treatment, although we cannot predict the direction of change. Finally, we expect structural and MRS findings to be associated with clinical improvement.

## Materials and methods

### Participants

Twelve inpatients with TRD were consecutively recruited from the Mood Disorders Inpatient Unit of Bellvitge University Hospital, Barcelona, Spain. At inclusion, all the patients met criteria for a major depressive episode according to the Diagnostic and Statistical Manual of Mental Disorders (DSM-IV-TR)^26^ and the independent evaluation performed by two senior psychiatrists (MU and NC) using the Structured Clinical Interview for DSM-IV Axis I Disorders–Clinician Version.^27^ Inter-rater agreement was 100%. All the patients underwent a comprehensive clinical assessment before ECT, including a physical examination, routine blood sampling, EKG and a chest x-ray. All the patients were under medication, which was kept constant throughout the ECT protocol. A list of concurrent pharmacological treatment is provided in Supplementary Table 1.

The control group was made up of 10 subjects matched for age, gender and years of education. Each healthy control underwent the non-patient version of the Structured Clinical Interview for DSM-IV Axis I Disorders^28^ to exclude any Axis I psychiatric disorders and the use of psychotropic medication. For both the groups, exclusion criteria included: (i) the presence or past history of a severe medical or neurological disorder, (ii) contraindication to MRI scanning or abnormal MRI upon visual inspection and (iii) a history of ECT during the previous 12 months.

The study was approved by the ethics committee of clinical research (CEIC) of Bellvitge University Hospital, and was performed in compliance with national legislation and the principles expressed in the Declaration of Helsinki. All the participants gave written informed consent after being provided with a detailed description of the study.

### Electroconvulsive therapy

Following our ECT unit protocol and national and international standards,^29, 30, 31^ all the patients were treated with bifrontotemporal, brief pulse (0.5–1 ms) ECT, using a Thymatron System IV device (Somatics, Lake Bluff, IL, USA). Anesthesia was induced with intravenous thiopental (2–2.5 mg kg^−1^) and succinylcholine (0.5 mg kg^−1^) was used for muscle paralysis. The patients were pre-oxygenated and then manually ventilated during the duration of anesthesia. Initial electrical dose was determined by the half-age method^32^ and subsequent dosing was determined according to seizure morphology adequacy. The psychotropic medications were maintained unchanged throughout the ECT treatment course.

### Study protocol

The patients were scanned four times: 24–48 h before the first ECT session (MRI1—baseline), 24–48 h after the first ECT session (MRI2—early ECT effects), 24–48 h after the ninth ECT session (MRI3—intermediate ECT effects) and 2 weeks after the completion of the ECT course (MRI4—long-lasting ECT effects). The control subjects were scanned twice, 5 weeks apart. Moreover, all the patients underwent a weekly clinical assessment using the Hamilton Rating Scale for Depression (HRSD-21 items).^33^

### MRI and MRS acquisition and preprocessing

All the MR studies were performed in a Philips Achieva 3.0 Tesla magnet scanner (Philips Healthcare, Best, The Netherlands), equipped with an eight-channel phased-array head coil provided by the manufacturer to obtain three-dimensional anatomical images and proton (^1^H) MR spectroscopy.

#### Three-dimensional anatomical images

High-resolution anatomical images were obtained using a sagittal T1-weighted three-dimensional fast spoiled gradient sequence. A total of 160 slices were acquired with repetition time=8.1 ms; echo time=3.7 ms; flip angle=8°, field of view=240 × 240 mm; matrix size 256 × 256 pixels, in-plane resolution=0.94 × 0.94 mm^2^; slice thickness=1 mm.

Structural MRI data were processed on a Microsoft Windows platform using technical computing software (MATLAB 7.14; The MathWorks, Natick, MA, USA) and Statistical Parametric Mapping (SPM12; The Welcome Department of Imaging Neuroscience, London, UK). First, the images were visually inspected by experienced members of the research team for the presence of any artifacts preventing further analyses. Subsequent image preprocessing was performed at the individual level, and consisted of an initial rigid-body (that is, translations and rotations) within-subject co-registration to the first scan of the series to ensure good starting estimates.

This was followed by pairwise longitudinal registrations between the scans from different time points using symmetric diffeomorphic modeling^34^ to assess volumetric change across the ECT course. This step incorporated a bias field correction to minimize the effects of intensity inhomogeneities on further preprocessing.^35^ This algorithm generated, for each image pair, an average image (that is, the average of the MRI images from the two time points) and a Jacobian difference map (that is, a map of the regional volume change between the two time points). The average image was segmented with the New Segment algorithm^36^ to isolate GM voxels and, to specifically identify GM volume changes, GM voxels from the segmented average image were multiplied by the volume change map (so we focused on longitudinal volume changes of GM regions). Next, we obtained a specific template of our study sample (in Montreal Neurological Institute (MNI) space) using a Diffeomorphic Anatomical Registration Through Exponentiated Lie Algebra algorithm,^37, 38^ which was used to spatially normalize the GM volume change maps before between-group comparisons. Importantly, no modulation (that is, volume restoration) was performed after the normalization to the MNI template. Finally, the resultant images were smoothed with an 8 mm full-width at half maximum isotropic Gaussian Kernel.

#### ^1^H MR spectroscopy

The spectra were acquired using a single-voxel Point-Resolved Spectroscopy sequence with TR, 2000 ms; TE, 32 ms and 256 averages. In addition, unsuppressed water scans (19 averages) were acquired alongside each scan. Total scan duration was 9 min 8 s. A total of 2048 data points were collected over a spectral width of 2000 Hz. The volume of interest acquired in each participant was adjusted to individual left hippocampus anatomy. Mean (s.d.) voxel dimensions were 27.9 (3.8) mm × 13.4 (1.7) mm × 10.4 (1.6) mm and the total mean volume size 4.0 (1.6) ml. Repositioning accuracy was optimized by use of the Philips SameScan auto-position tool and visual matching with baseline geometry in the three orthogonal planes. Shimming and tuning were carried out via automated procedures before acquisition. Water signal was suppressed with selective water signal inversion.

The spectra analysis was performed off-line with the use of jMRUI software (available through the MRUI Project, http://www.mrui.uab.es).^39, 40^ Fitted areas of the resonances of interest were calculated in the water-suppressed spectrum by using the Advanced Magnetic Resonance (AMARES) algorithm as described previously.^41^ Assignment of the resonances of interest included NAA at 2.02 p.p.m., glutamate and glutamine (Glx) at 2.35 p.p.m., creatine plus phosphocreatine (Cr) at 3.03 p.p.m. and choline and other trimethylamine-containing compounds (Cho) at 3.20 p.p.m.^42^ For spectra quality, minimum values of metabolite SNR > 10 and unsuppressed water peak linewidth < 8 Hz were used. In addition, each water-suppressed spectrum was quality checked by visual inspection to detect artifacts that would adversely affect the quality of the results such as large baseline distortions, exceptionally broadened metabolite peaks, insufficient removal of the water line, large phase errors and presence of signals originating from outside the voxel.

### Statistical analyses

Sociodemographic and clinical data were analyzed with SPSS v.21 (SPSS, Chicago, IL, USA) using nonparametric tests.

To assess ECT effects on GM volumes, we first performed a whole-brain voxel-wise volumetric comparison between volume change maps of patients with TRD and healthy controls in SPM. Importantly, to facilitate the interpretation of our findings, we exclusively examined changes relative to baseline. Specifically, we estimated three two-sample *t*-test models, corresponding to the analysis of three time-point differences (MRI1–MRI2, MRI1–MRI3 and MRI1–MRI4). Age and gender were included as confounding covariates. Statistical significance was set at *P*<0.05 (two-sided), family-wise error corrected for multiple comparisons.

Next, we assessed the structural covariance between the regions of significant longitudinal volume change. Specifically, we extracted the voxel values from the peak coordinates of the above analyses (representing the regional volume change) and obtained the correlations between such longitudinal changes. We used Spearman's *ρ* correlation coefficients, with a statistical significance threshold of *P*<0.05 (two-sided) after Bonferroni correction for multiple comparisons. These analyses were performed in SPSS v.21.

A Wilcoxon signed-rank test analysis was used to assess across-time changes in hippocampal metabolite concentrations. Cross-sectional between-group differences in this imaging assessment were assessed with Mann–Whitney *U*-tests. In addition, the relationship between GM volume changes (extracted from the peak coordinates of the above analyses) and hippocampal metabolite concentration changes was explored using Spearman's *ρ* correlation coefficients. In such analyses, we used a statistical significance threshold of *P*<0.05 (two-sided) after a Bonferroni correction for multiple comparisons when appropriate. All these analyses were performed in SPSS v.21.

The association between clinical response and GM volume changes was assessed using multiple regression analyses. Clinical response, as measured by the percentage of change in HRSD scores, was included as an independent predictor in an SPM multiple regression model to evaluate its effect on regional GM volume changes. Importantly, this analysis was focused on the pattern of significant results derived from the above whole-brain regional volume comparison. Age and gender were included as confounding covariates. Statistical significance was set at *P*<0.05 (two-sided), family-wise error corrected for multiple comparisons across all in-mask voxels (that is, using small-volume correction procedures). The relationship between clinical response and hippocampal metabolite concentration changes was assessed using Spearman's *ρ* correlation coefficients, with a statistical significance threshold of *P*<0.05 (two-sided) after Bonferroni correction for multiple comparisons. These last analyses were performed in SPSS v.21.

## Results

### Sociodemographic and clinical characteristics

Twelve patients with TRD received an acute bitemporal ECT course. The mean number of sessions per week (±s.d.) was 2.43 (±0.38), averaging 11 ECT sessions per patient (mean ECT sessions±s.d.=11.08±1.50). The mean HRSD score (±s.d.) before ECT initiation was 31.25 (9.21) and the mean HRSD score (±s.d.) after the completion of the ECT was 2.92 (2.54). At the end of the treatment, all the patients fulfilled clinical response criteria (reduction >50% in HRSD score) and all but one were in clinical remission (HRSD<8). Moreover, the reduction in depression severity (HRSD score) between MRI1 and MRI4 assessments was significant according to a Wilcoxon signed-rank test (*z*=−3.062; *P*=0.002). Importantly, this significant reduction in depression severity was already observed at MRI3 (Wilcoxon signed-rank test, *z*=−3.059; *P*=0.002). Sociodemographic and clinical characteristics of the study samples are summarized in Table 1.

### Neuroimaging analyses

#### Structural MRI

**Figure 1:**
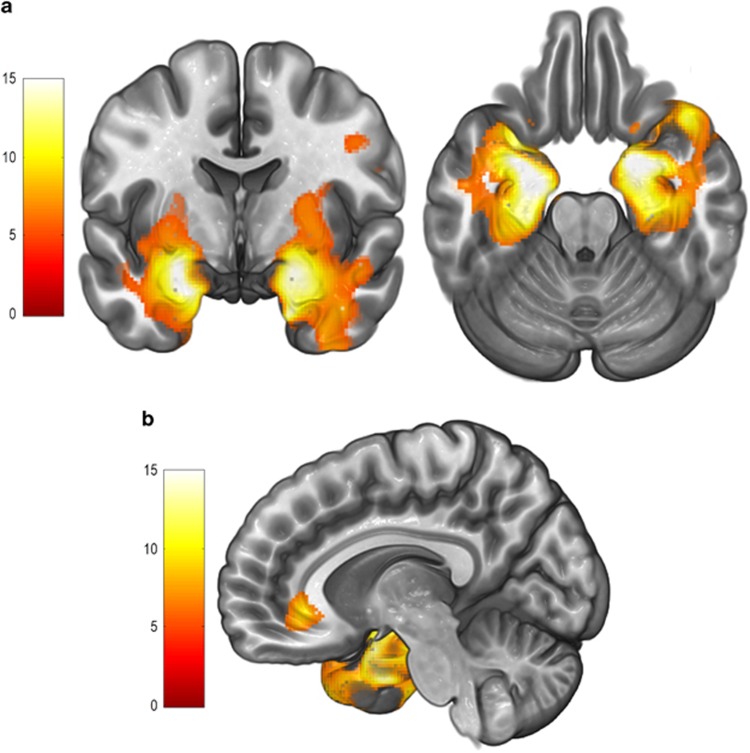
(**a**) Volume changes in bilateral MTL (including the bilateral amygdala, hippocampus and parahippocampus) between MRI1 and MRI3, overlaid on coronal and axial slices in MNI-normalized space. The cluster of significant findings extended to other areas such as the insula, the striatum or the inferior temporal cortex, although the results on these regions were much less defined and less significant in relation with findings in the MTL. Left hemisphere is depicted on the left. (**b**) Volume change in the right perigenual anterior cingulate cortex (rPgACC) between MRI1 and MRI3, overlaid on a sagittal slice in MNI-normalized space. Volume increases in the right temporal pole (extending from the cluster depicted in **a**) may be also appreciated. Color bar represents *t*-value. MNI, Montreal Neurological Institute; MRI, magnetic resonance image; MTL, medial temporal lobe.

GM volume change from MRI1 to MRI2 did not significantly differ between the groups. By contrast, in comparison with healthy controls, from MRI1 to MRI3, TRD patients showed a significant GM volume increase in: (i) the medial temporal lobe (MTL), including bilateral amygdala, hippocampus and parahippocampal cortex and (ii) the right perigenual anterior cingulate cortex (rPgACC). In addition, the GM volume increase in the MTL remained significant at MRI4 in comparison with MRI1. These results are summarized in Table 2 and Figure 1. At an exploratory level, we observed that these same regions did also significantly increase from MRI2 to both MRI3 and MRI4, however, to facilitate the interpretation of our findings and since the first significant clinical and imaging changes were observed at MRI3, subsequent imaging analyses were centered on the comparison of this time point with baseline.

For reference, in Supplementary Material, we provide a baseline voxel-based morphometry analysis comparing MDD patient group vs controls before ECT. No significant between-group differences were observed at the selected significance threshold.

#### Structural covariance

Interestingly, we observed significant correlations between the left and right MTL MRI1–MRI3 volume changes (*ρ*=0.699, *P*=0.011) and between left MTL and rPgACC volume changes (*ρ*=0.636, *P*=0.026). As such, this reflects the existence of structural covariance in the volume change of these structures.

#### ^1^H MR spectroscopy

**Figure 2:**
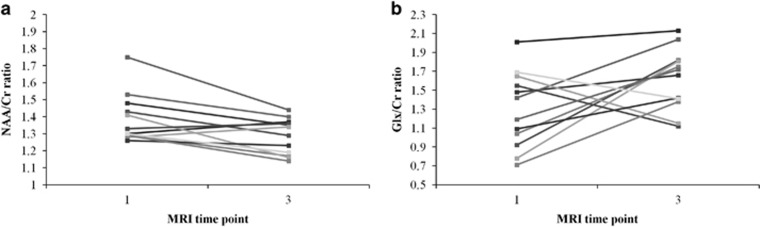
(**a**) Individual hippocampal NAA/Cr ratio change between MRI1 and MRI3 in patients with TRD. (**b**) Individual hippocampal Glx/Cr ratio change between MRI1 and MRI3 in patients with TRD. Cr, creatine plus phosphocreatine; Glx, Glutamate+Glutamine; MRI, magnetic resonance image; NAA, *N*-acetylaspartate.

An example spectrum and fitting is provided in Supplementary Figure 1. A Wilcoxon signed-rank test on hippocampal metabolite concentrations revealed that NAA/Cr ratio significantly decreased after the ninth ECT session (MRI3; *z*=−2.432, *P*=0.015), whereas the Glx/Cr ratio increased at a trend level at this same time point (*z*=−1.961, *P*=0.050; see Figure 2). Cho/Cr ratio did not significantly differ between time points. Likewise, MRS data from healthy controls did not differ between time points. Moreover, the between-group (patient vs control) comparison of pre- and post-ECT MRS assessments did not detect any significant difference for any metabolite.

**Figure 3:**
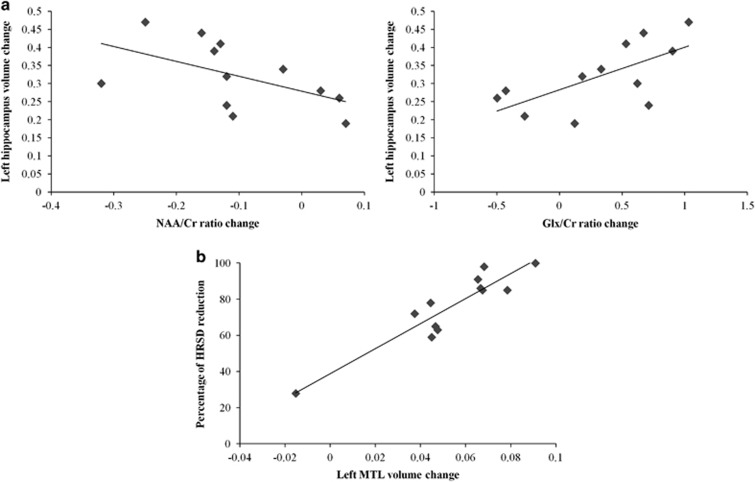
(**a**) Scatter plots depicting the relationship between regional gray matter (GM) volume change (adjusted values) in the left hippocampus and hippocampal NAA/Cr ratio (left) and Glx/Cr ratio (right) changes. (**b**) Scatter plot depicting the relationship between regional GM volume change (adjusted values) in the left medial temporal lobe (MTL) and clinical response (percentage of HRSD reduction) between MRI1 and MRI3. Statistical significance of the correlation held after excluding the subject with the lowest values in the two variables. Cr, creatine plus phosphocreatine; Glx, Glutamate+Glutamine; HRSD, Hamilton Rating Scale for Depression; MRI, magnetic resonance image; NAA, *N*-acetylaspartate.

Importantly, in the patient group, we observed a significant correlation between the left hippocampus volume change and the hippocampal NAA/Cr ratio change (*ρ*=−0.706, *P*=0.010), as well as a trend level correlation between the left hippocampus volume change and the hippocampal Glx/Cr ratio change (*ρ*=0.629, *P*=0.028, nonsignificant after Bonferroni correction for multiple comparisons; see Figure 3a).

#### Relationship with clinical change

An SPM multiple regression analysis including clinical response from MRI1 to MRI3 as the independent predictor and the voxel-wise MRI1–MRI3 GM volume change within the cluster of significant findings from the previous structural comparisons as the dependent variable (that is, using small-volume correction procedures) revealed a significant (*t*=7.26; *P*=0.047) positive association between clinical improvement and regional GM volume increase in the left MTL (*x*=−39, *y*=−12, *z*=−14; see Figure 3b).

Conversely, using Spearman correlation analyses in SPSS, we observed that metabolite concentrations in the hippocampus were not related with clinical improvement.

## Discussion

To our knowledge, this is the first longitudinal study evaluating brain changes throughout the course of an acute bitemporal ECT protocol in patients with TRD by means of different neuroimaging approaches (that is, structural longitudinal voxel-wise analyses and magnetic resonance spectroscopy). Specifically, we observed regional volume increases in the MTL and the rPgACC after ECT, as well as an association of left MTL volume increase with a hippocampal NAA/Cr concentration decrease and a hippocampal Glutamate+Glutamine (Glx/Cr) concentration increase. Likewise, left MTL volume increase was associated with clinical improvement.

Our structural findings concur with previous GM volume studies reporting significant volume increases in these same areas after ECT.^2, 3, 4, 5, 6, 7, 8, 9, 10, 11, 12^ Indeed, these brain regions have consistently been cited as being critical in accounting for depressive symptomatology and disease remission in prevailing neurobiological models of depression.^43, 44^ Interestingly, volume increases in the left and right MTL were significantly correlated, and we also observed a correlation between volume increases in the left MTL and the rPgACC.

These results suggest the existence of ECT-induced structural covariance (that is, volume correlation between distant brain regions) between these structures. Indeed, structural covariance may rely on structural neuroplasticity,^45, 46^ and, therefore, the findings from our study (that is, more significant changes in the MTL than in the rPgACC and structural covariance between these structures) may indicate that the molecular-level mechanisms of ECT action are probably similar across different brain regions, differing in their magnitude as the distance from the energy source increases. Nevertheless, according to this idea, it remains to be established why structural effects are only observed in limbic structures and not in neocortical regions, closer to the electrode location. In this sense, however, it is important to highlight that structural plasticity is much more frequently observed in limbic regions than in the prefrontal, motor and sensory cortices.^47, 48, 49^

Notwithstanding the above ideas on the link between ECT and structural plasticity, it should be noted that while hippocampal neurogenesis and other forms of neural plasticity have consistently shown a positive effect on learning and memory capacity,^50, 51, 52^ ECT is undoubtedly associated with significant impairments in memory and executive functions.^53, 54^ Importantly, hippocampal volume increases in our subjects were related to an NAA/Cr ratio decrease and a trend-level Glx/Cr ratio increase. As NAA levels are considered to be associated with healthy neural function,^55^ such NAA decreases seem to indicate a significant ECT-induced neural malfunction at the hippocampal level, which would be in agreement with the above-mentioned cognitive side-effects of ECT. In accordance with our findings, one previous study has also identified hippocampal NAA concentration decreases after ECT.^20^ By contrast, three other studies identified no significant changes in hippocampal NAA concentration, although these studies were performed in 1.5T magnets, which provide a worse signal-to-noise ratio for detecting metabolite concentrations,^21, 22^ or acknowledged low-quality spectra for the hippocampal region.^9^ The combination of hippocampal NAA concentration decrease with a regional volume increase may suggest that ECT-induced changes are due to neuronal swelling or of non-neural origin (for example, angiogenesis). These two options are discussed below.

Regarding Glx increases, it is important to emphasize that, as opposed to previous studies focused on the ACC, the dorsal lateral prefrontal cortex or the amygdala,^18, 20, 24, 25^ we did not observe a decreased Glx/Cr ratio in our TRD patients before treatment initiation. Therefore, our results do not seem to support the idea that Glx increases were a consequence of an ECT-induced normalization in hippocampal Glx levels. They may be better interpreted within the framework of the neuroinflammatory hypothesis of ECT action.^8, 16^ Indeed, it is well known that excessive glutamate levels may lead to neuronal swelling, secondary to the influx of cations and water.^23^ In addition, although neuroinflammation has been typically associated with ECT cognitive side-effects,^16^ recent research suggests that in TRD patients undergoing neurosurgery for implanting a deep brain stimulation electrode in the subgenual ACC, early clinical improvement (that is, before the actual initiation of the deep brain stimulation treatment) was associated with regional neuroinflammation and attenuated by anti-inflammatory treatment (that is, NSAIDs).^56^ Therefore, our findings suggest the existence of a common neurobiological mechanism accounting for ECT-induced clinical improvement and cognitive side-effects. However, contrary to our results, a recent study reported hippocampal Glx concentration increases at baseline that were reduced after ECT.^20^ Nevertheless, these findings were exclusively observed in the left hippocampus, while most patients received a right unilateral treatment. In this sense, it is well known that current density in unilateral ECT is substantially larger in the ipsilateral than in the contralateral hemisphere.^57^ Therefore, further research is warranted to better characterize and understand the contralateral effects of unilateral treatment protocols.

In addition, besides the direct link with neuroinflammation, Glx increases may also boost angiogenesis to satisfy putative subsequent increases in metabolic demand, which may in turn lead to an increase in the permeability of the blood–brain barrier^17^ and finally to a local swelling of the adjacent brain tissue.^16^ Importantly, hippocampal angiogenesis has previously been postulated as a candidate neurobiological correlate of ECT mechanism of action in pre-clinical research.^58^ Insofar angiogenesis may be considered a form of neuroplasticity,^10, 15^ our findings reconcile the neuroplasticity and neuroinflammatory hypotheses of ECT action. Furthermore, other neuroplasticity mechanisms, such as synaptogenesis, have been shown to be induced after ketamine administration, which is supposed to increase glutamate neurotransmission.^59^

Regarding the relationship between our imaging results and clinical improvement, it seems that only structural changes in the MTL, but not in the rPgACC, were significantly associated with symptom reduction. We think these results give further support to the above-mentioned idea of a proportional decrease of ECT effect with increasing distance from the electrode. Moreover, the lack of a direct association between our metabolic results and clinical improvement may also imply that NAA and Glx changes only partially account for ECT-induced changes at the molecular level. Other molecular routes such as those involving glial-fibrillary-acidic-protein and p11 expression^56^ should be explored in further research. However, it should be noted that we observed an indirect association between metabolic changes and clinical improvement through volumetric change. Therefore, further analyses with other statistical approaches such as structural equation modeling are warranted to ascertain as to what extent the metabolic changes observed here may be indirectly associated with clinical improvement. Finally, the lateralization of our correlation between imaging changes and clinical improvement to the left hemisphere seems to partially account for the previously described superiority of bilateral ECT in front of right unilateral approaches in terms of clinical response.^60, 61, 62^

This study has a number of limitations. First, given the small number of patients included, the results should be considered preliminary and, therefore, replication with a larger sample is warranted. Nevertheless, our sample was carefully characterized with weekly clinical assessments and four multimodal neuroimaging assessments covering the entire course of the treatment, which allowed for a comprehensive characterization of different neurobiological changes and their association with clinical response. Likewise, our results should not necessarily generalize to patient samples undergoing different ECT protocols (for example, unilateral treatments). Finally, we cannot determine what effect, if any, concurrent pharmacological treatment had on our findings, although in an attempt to minimize this confounding effect, pharmacological treatment was not modified throughout the entire ECT course. Importantly, we did not observe any structural or metabolic differences between patient and control groups in pre-ECT comparisons. In this sense, it should be noted that although pharmacological treatment with selective serotonin reuptake inhibitors has been related with the induction of neural plasticity,^63^ neuroinflammatory and volumetric changes in limbic regions have been seldom reported after successful pharmacological trials.^64^ This seems to suggest that these latter effects may be predominantly observed after ECT, therefore providing a specific clinical benefit to patients with TRD.

In conclusion, both neural plasticity and neuroinflammation may be induced by ECT and partially account for its clinical effectiveness. The nature of the changes seems to be similar across different brain regions, although the magnitude of the effects may decrease as the distance from the energy source rises. Our results also indicate that structural, but not metabolic, changes in the left medial temporal lobe are the best neuroimaging biomarkers of ECT-induced clinical response in TRD. Further research is needed to ascertain whether a common neurobiological underpinning may account for both positive and negative consequences of ECT.

## Figures and Tables

**Table 1:** Sociodemographic and clinical characteristics of the study samples

*Sociodemographic and clinical variables*^a^	*Patients with TRD (*n=*12)*	*Comparison participants (*n=*10)*	*Statistic*^b^	P*-value*
Age, years: mean (s.d.)	59.17 (8.02)	54.4 (8.37)	−1.454	0.159
Gender, male: *n* (%)	6 (50)	5 (50)	0	1
Education, years: mean (s.d.)	9.75 (5.91)	12.44 (3.39)	−1.253	0.219
Psychotic symptoms: *n* (%)	5 (41.7)			
Comorbid anxiety disorder: *n* (%)	3 (25)			
Age at onset, years: mean (s.d.)	40.58 (19.00)			
Duration of current episode, weeks: mean (s.d.)	61.75 (134.46)			
Number episodes: mean (s.d.)	4.08 (2.71)			
HRSD1^c^: mean (s.d.)	31.25 (9.21)			
HRSD2: mean (s.d.)	28.50 (9.52)			
HRSD3: mean (s.d.)	6.75 (5.17)			
HRSD4: mean (s.d.)	2.92 (2.54)			
MSM: mean (s.d.)	9.33 (1.15)			

*Drugs: %*				
Antidepressant	100			
Antipsychotics	75			
Lithium	16.7			
Anxiolytics	50			
Previous ECT: %	16.7			

Abbreviations: ECT, electroconvulsive therapy; HRSD, Hamilton Rating Scale for Depression; MSM, Maudsley Staging Model; TRD, treatment-resistant depression.

a No significant differences were observed between groups in any of the variables.

b Mann–Whitney *U*-test for continuous variables, *χ*^2^ test for categorical variables.

c This number indicates the time point corresponding to the HRSD score. 1—Score in the first neuroimaging assessment; 2—Score in the second neuroimaging assessment; 3—Score in the third neuroimaging assessment; and 4—Score in the fourth neuroimaging assessment.

**Table 2:** Brain areas showing gray matter volume increases in patients with treatment-resistant depression

	*x*	*y*	*z*	t*-value*	P*-value*^a^FWE (family-wise error) corrected for multiple comparisons.	*Anatomical location*
*MRI1*–*MRI3*						
Left MTL (12 137 voxels)	−26	−3	−21	14.92	<0.001	Left amygdala
	−24	−6	−23	14.28	<0.001	Left hippocampus
	−21	−7	−26	13.36	<0.001	Left parahippocampus
Right MTL (18 120 voxels)	21	0	−20	13.15	<0.001	Right amygdala
	23	−1	−20	13.01	<0.001	Right hippocampus
	21	2	−23	13.35	<0.001	Right parahippocampus
	8	35	−5	8.42	<0.001	rPgACC (553 voxels)

*MRI1*–*MRI4*						
Left MTL (10 985 voxels)	−26	−4	−14	12.81	<0.001	Left amygdala
	−26	−7	−14	11.85	<0.001	Left hippocampus
	−26	−7	−14	11.85	<0.001	Left parahippocampus
Right MTL (15 460 voxels)	23	−4	−18	13.81	<0.001	Right amygdala
	23	−6	−21	14.02	<0.001	Right hippocampus
	26	−13	−26	13.97	<0.001	Right parahippocampus

a Abbreviations: MRI, magnetic resonance image; MTL, medial temporal lobe; rPgACC, right perigenual anterior cingulate cortex. *x, y, z* coordinates are reported in standard Montreal Neurological Institute (MNI) space.
