# Supplementary material for: Brain volumetric and metabolic correlates of electroconvulsive therapy for treatment-resistant depression: a longitudinal neuroimaging study
Source: Transl Psychiatry. 2017 Feb 7;7(2):e1023–. doi: 10.1038/tp.2016.267 (PMC5438019; doi:10.1038/tp.2016.267)
Supplement: Supplementary Information [file tp2016267x1.docx]

**Supplementary Material**

**Table SM1.** Concurrent pharmacological regimen of patients with TRD

| **Subject** | | **SSRI**  **(mg/day)** | | **SNRI**  **(mg/day)** | | **NaSSA**  **(mg/day)** | | **TCA**  **(mg/day)** | | **SGA**  **(mg/day)** | | **Lithium**  **(mg/day)** | | **BZD**  **(mg/day)** |
| --- | --- | --- | --- | --- | --- | --- | --- | --- | --- | --- | --- | --- | --- | --- |
| **1** | Fluoxetine  (20) | | Venlafaxine  (300) | |  | |  | | Olanzapine  (10) | |  | |  | |
| **2** |  | | Venlafaxine  (300) | |  | |  | | Olanzapine  (20) | |  | | Lormetazepam  (2) | |
| **3** | Paroxetine  (20) | | Venlafaxine  (300) | |  | |  | |  | | Lithium  (1200) | |  | |
| **4** |  | | Duloxetine  (90) | | Mirtazapine  (30) | |  | | Quetiapine  (150) | |  | |  | |
| **5** | Fluoxetine  (60) | |  | |  | |  | | Quetiapine  (150) | |  | |  | |
| **6** |  | | Duloxetine  (120) | | Mirtazapine  (30) | |  | |  | |  | | Flunitrazepam  (1) | |
| **7** |  | | Venlafaxine  (225) | |  | | Imipramine  (100) | |  | |  | | Lormetazepam  (2) | |
| **8** |  | | Venlafaxine  (300) | |  | |  | | Olanzapine  (20) | |  | | Clonazepam  (2) | |
| **9** |  | | Duloxetine  (120) | | Mirtazapine  (45) | |  | | Olanzapine  (5) | |  | |  | |
| **10** | Paroxetine  (60) | | Duloxetine  (60) | |  | |  | | Quetiapine  (150) | |  | | Flunitrazepam  (1) | |
| **11** | Sertraline  (100) | |  | | Mirtazapine  (30) | | Imipramine  (250) | | Aripiprazole  (5) | |  | | Lormetazepam  (2) | |
| **12** | Fluoxetine  (60) | | Venlafaxine  (150) | |  | | Imipramine  (200) | | Olanzapine  (5) | | Lithium  (600) | |  | |

*SSRI*, Serotonine-Selective-Reuptake-Inhibitor; *SNRI*, Serotonine-Noradrenaline-Reuptake-Inhibitor; *NaSSa*, Noradrenergic and Specific Serotonergic Antidepressant; *TCA*, Tricyclic Antidepressants; *SGA*, Second Generation Antipsychotics; *BZD*, Benzodiazepines

**Baseline voxel-brain morphometry analysis**

*MRI preprocessing*

Baseline (MRI1) data were processed on a Microsoft Windows platform using technical computing software (MATLAB 7.14; The MathWorksInc, Natick, Mass) and Statistical Parametric Mapping (SPM12; The Welcome Department of Imaging Neuroscience, London, UK). Specifically, imaging data pre-processing followed the voxel-based morphometry-DARTEL^1^ pipeline, including tissue segmentation (using the New Segment algorithm), normalization to the Montreal Neurological Institute (MNI) coordinate system, modulation, and smoothing with an 8mm full-width at half-maximum (FWHM) isotropic Gaussian kernel.

*Statistical analyses and Results*

We performed an exploratory whole-brain and voxel-wise two-sample *t*-test comparison in SPM (MDD patients vs. controls), with age and gender as confounding covariates. Statistical significance was set at p<0.05 (two-sided), Family-Wise error (FWE) corrected for multiple comparisons. No significant between-group differences were observed.

*References:*

1. Ashburner J. A fast diffeomorphic image registration algorithm. *Neuroimage* 2007; **38**: 95-113.

**
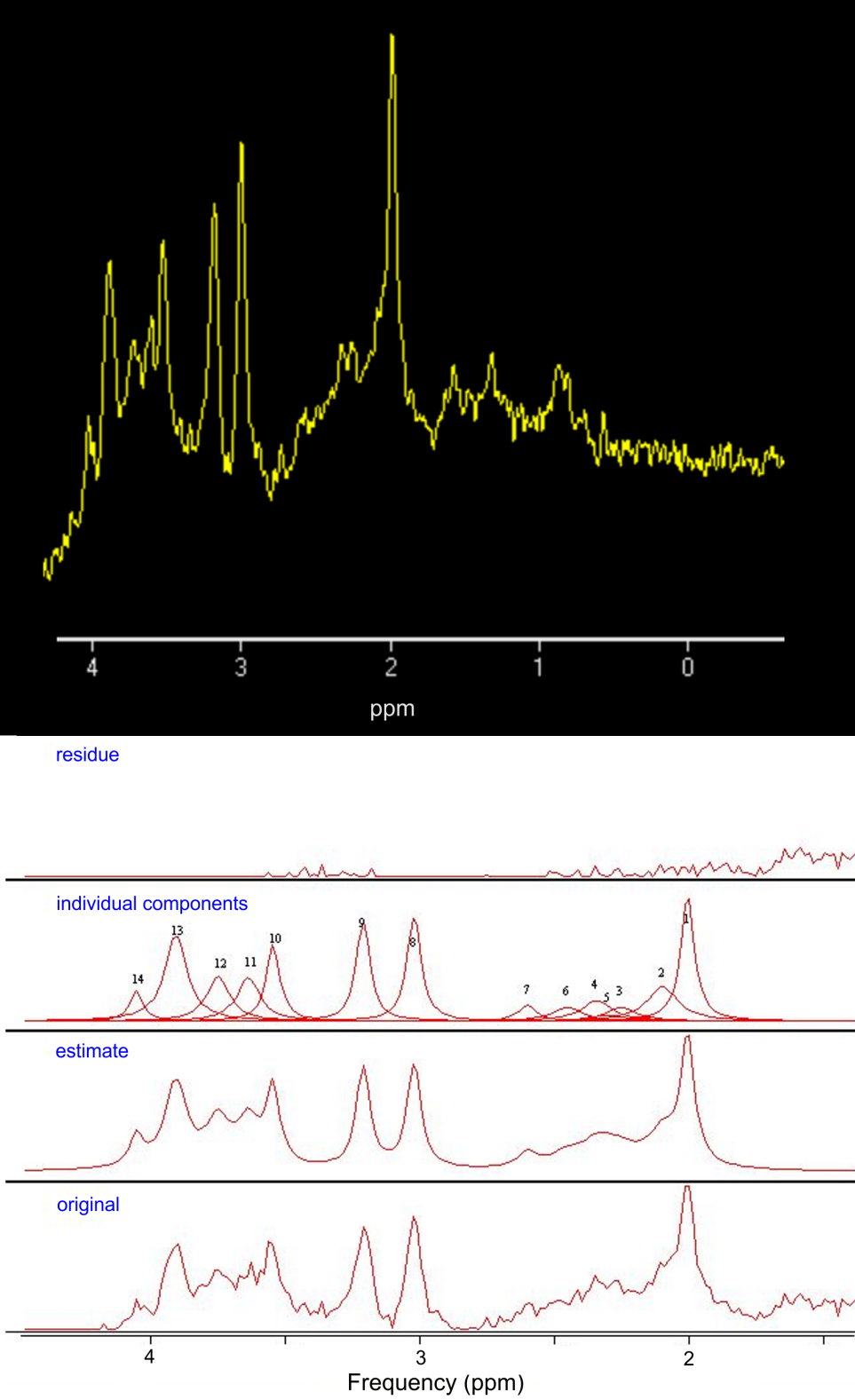
**

**Figure SM1.** Representative spectrum acquired in the hippocampus of a participant. *Top*:  original display *Bottom*: spectrum fitting obtained with the jMRUI software.
